# Elucidating the mechanisms of α-Synuclein-lipid interactions using site-directed mutagenesis

**DOI:** 10.1016/j.nbd.2024.106553

**Published:** 2024-06-03

**Authors:** Abid Ali, Aidan P. Holman, Axell Rodriguez, Luke Osborne, Dmitry Kurouski

**Affiliations:** a Department of Biochemistry and Biophysics, Texas A&M University, College Station, TX 77843, United States; b Department of Entomology, Texas A&M University, College Station, TX 77843, United States; c Department of Biomedical Engineering, Texas A&M University, College Station, TX 77843, United States

**Keywords:** a-Synuclein, Fatty acids, AFM-IR, Toxicity

## Abstract

α-Synuclein (α-syn) is a small protein that is involved in cell vesicle trafficking in neuronal synapses. A progressive aggregation of this protein is the expected molecular cause of Parkinson’s disease, a disease that affects millions of people around the world. A growing body of evidence indicates that phospholipids can strongly accelerate α-syn aggregation and alter the toxicity of α-syn oligomers and fibrils formed in the presence of lipid vesicles. This effect is attributed to the presence of high copies of lysines in the N-terminus of the protein. In this study, we performed site-directed mutagenesis and replaced one out of two lysines at each of the five sites located in the α-syn N-terminus. Using several biophysical and cellular approaches, we investigated the extent to which six negatively charged fatty acids (FAs) could alter the aggregation properties of K10A, K23A, K32A, K43A, and K58A α-syn. We found that FAs uniquely modified the aggregation properties of K43A, K58A, and WT α-syn, as well as changed morphology of amyloid fibrils formed by these mutants. At the same time, FAs failed to cause substantial changes in the aggregation rates of K10A, K23A, and K32A α-syn, as well as alter the morphology and toxicity of the corresponding amyloid fibrils. Based on these results, we can conclude that K10, K23, and K32 amino acid residues play a critical role in protein-lipid interactions since their replacement on non-polar alanines strongly suppressed α-*syn*-lipid interactions.

## 1. Introduction

Parkinson’s disease (PD) is a severe disease that is projected to strike 12 million people by 2040 worldwide. (Chen, 2010) *Postmortem* analysis of brains of PD patients revealed a substantial loss of dopaminergic (DA) neurons in the substantia nigra pars compacta (SNc), as well as other regions of the brain. (Hawkes et al., 2007; Braak et al., 2003; Braak et al., 2004) PD is also characterized by the presence of Lewy bodies (LBs) in midbrain, hypothalamus and thalamus. (Krack et al., 2019; Hoffmann et al., 2019; Vogiatzi et al., 2008) These intracellular formations are primarily composed of α-synuclein (α-syn) fibrils and fragments of cell membranes. (Luk et al., 2012a; Luk et al., 2012b; Shahmoradian et al., 2019) Numerous in vitro and in vivo experiments confirmed that α-syn, a small protein involved in cell vesicle trafficking in neuronal clefts, can aggregate in the presence of lipid bilayers forming highly toxic oligomers and fibrils. (Fecchio et al., 2013; Srinivasan et al., 2013) Based on these findings, a progressive aggregation of α-syn is the expected underlying molecular cause of PD. (Davie, 2008; Harris et al., 2009)

The molecular composition of lipid membranes, as well as the protein-to-lipid ratio, alter the rates of α-syn aggregation. For instance, it was found that anionic lipids accelerated α-syn aggregation, whereas this effect was not evident for zwitterionic lipids. (Dou et al., 2023; Galvagnion, 2017; Galvagnion et al., 2016; Galvagnion et al., 2015; Chen et al., 2015) Furthermore, at low concentrations of lipid vesicles, a significant increase in the rate of α-syn aggregation was found. However, with a subsequent increase in the concentration of lipid vesicles, a decrease in the rate of protein aggregation was observed. (Galvagnion et al., 2015) Galvagnion and co-workers hypothesized that the former observation was due to lipid-facilitated protein-protein interactions that took place on the vesicular surfaces. (Galvagnion et al., 2015) Consequently, the latter was attributed to a drastic increase in the lipid surface areas that minimized the probability of protein-protein interactions and, consequently, the rate of α-syn aggregation. (Galvagnion, 2017) It was also found that the chemical structure of lipids that were present in the vesicles not only influenced the rate of protein aggregation, but also changed the structure and toxicity of α-syn oligomers and fibrils formed in the presence of such lipids. (Dou et al., 2023; Dou and Kurouski, 2022; Dou et al., 2021) For example, structurally different oligomers and fibrils were formed by α-syn in the presence of phosphatidylcholine (PC) and phosphatidylserine (PS). (Dou et al., 2023) Furthermore, these aggregates exerted higher cell toxicity in N27 rat dopaminergic cells compared to α-syn fibrils formed in the lipid-free environment. (Dou et al., 2023) Previously used NMR and fluorescence methods revealed mechanisms of such lipid-determined changes in the structure of α-syn aggregates. (Giasson et al., 2001; Ueda et al., 1993) It was found that polar headgroups of lipids interacted with lysine and glutamic acid residues on the N-terminus (aa 1–60) of a-syn. (Viennet et al., 2018) Protein-lipid interactions were also enhanced by fatty acids (FAs) of lipids with the central domain (aa 61–95) of α-syn, also known as the NAC domain. (Giasson et al., 2001; Ueda et al., 1993) Claessens group demonstrated that such interactions directly controlled by the charge and the size of lipid vesicles (Middleton and Rhoades, 2010; van Rooijen et al., 2009; Iyer and Claessens, 2019; Iyer et al., 2014; Iyer et al., 2016a; Iyer et al., 2016b; Stockl et al., 2008). Hannestad and co-workers found that α-syn not only aggregated on the surfaces of lipid vesicles, but strongly perturbed the integrity of lipid bilayers (Hannestad et al., 2020). Furthermore, Lee group found that α-syn could re-shape lipid bilayers (Jiang et al., 2018) It was shown that addition of α-syn to lipid vesicles composed of 1-palmitoyl-2-oleoyl-sn-glycero-3-phospho-(1′-rac-glycerol) (POPG) caused rapid re-shaping of these vesicles into micellar and bilayer tubules with 7 and 30 nm in diameter, respectively. These pieces of experimental evidence demonstrated that lipid-templated aggregation of α-syn was driven by both electrostatic and hydrophobic interactions between lipids and proteins.

If such interactions could be disrupted, lipid bilayers would fail to template the formation of highly toxic α-syn oligomers and fibrils. Based on the discussed above NMR results, we hypothesized that lysines located in the N terminus of α-syn play a key role in facilitating the protein-lipid interactions. Five sites within the N terminus have lysine (K) doublets: K10 and K12, K21 and K23, K32 and K34, K43 and K45, K58 and K60, as well as a single site with one K at the K6 position. In our study, we used site-directed mutagenesis approach to develop α-syn mutants in which one out of two lysines in each of the doubles is replaced with alanine (A). Thus, we made the following mutants: K10A, K23A, K32A, K43A, and K58A α-syn. We opted for alanine substitutions in each mutant because alanine preserves the main-chain conformation without affecting side chains beyond the β carbon. Additionally, it avoids introducing extreme electrostatic or steric effects.

Next, we determined the extent to which the aggregation properties of these mutants, as well as wild-type α-syn, can be altered by six negatively charged fatty acids (FAs), including saturated stearic acid (C18:0, STA) and polyunsaturated linoleic acid (C18:2, LA), α-linoleic acid (C18:3, ALA), dihomo-γ-linolenic acid (C20:3, DGLA), eicosapentaenoic acid (C20:5, EPA), and docosahexaenoic acid (C22:6, DHA), Fig. S1. The large number of FAs were utilized to exclude the possibility of the misinterpretation of our kinetic results. In our previous study, we showed that mutations in α-syn could change protein-lipid interactions with some FAs. For instance, PS with C14:0 FAs (1,2-dimyristoyl-*sn*-glycero-3-phospho-l-serine (DMPS)), C18:1 FAs (1,2-dioleoyl-*sn*-glycero-3-phospho-l-serine (DOPS)), C16:0 and C18:1 FAs (1-palmitoyl-2-oleoyl-*sn*-glycero-3-phospho-l-serine (POPS)), and C18:0 FAs (1,2-distearyl-*sn*-glycero-3-phospho-l-serine (DSPS)) accelerated the aggregation rate of WT α-syn. (Ali et al., 2023a) However, only DOPS, DMPS, and DSPS accelerated the rate of A53T α-syn, whereas POPS caused no changes in the aggregation properties of this mutant. Finally, DSPS accelerated, but DOPS, DMPS and POPS decelerated the aggregation rate of A30P α-syn. (Ali et al., 2023a) Recently reported results by Holman and co-workers also showed that FAs with different length and saturation could have different energies of interactions with the same protein. (Holman et al., 2023) Consequently, if only one or a few FAs are used to investigate the effect of K-to-A mutations, the expected differences in the aggregation properties of mutants could not be unambiguously assigned to the mutations themselves. The utilization of a large number of FAs with different lengths and saturation of alkyl chains is required to fully exclude any particular differences between any FA and α-syn. Although more laborious, such experiments allow for making a solid conclusion about the role of K-to-A mutations in α-*syn*-lipid interactions.

## 2. Results

### 2.1. Kinetic studies of K10A, K23A, K32A, K43A, K58A, and WT α-syn aggregation in the presence of different FAs

**Fig. 1.**
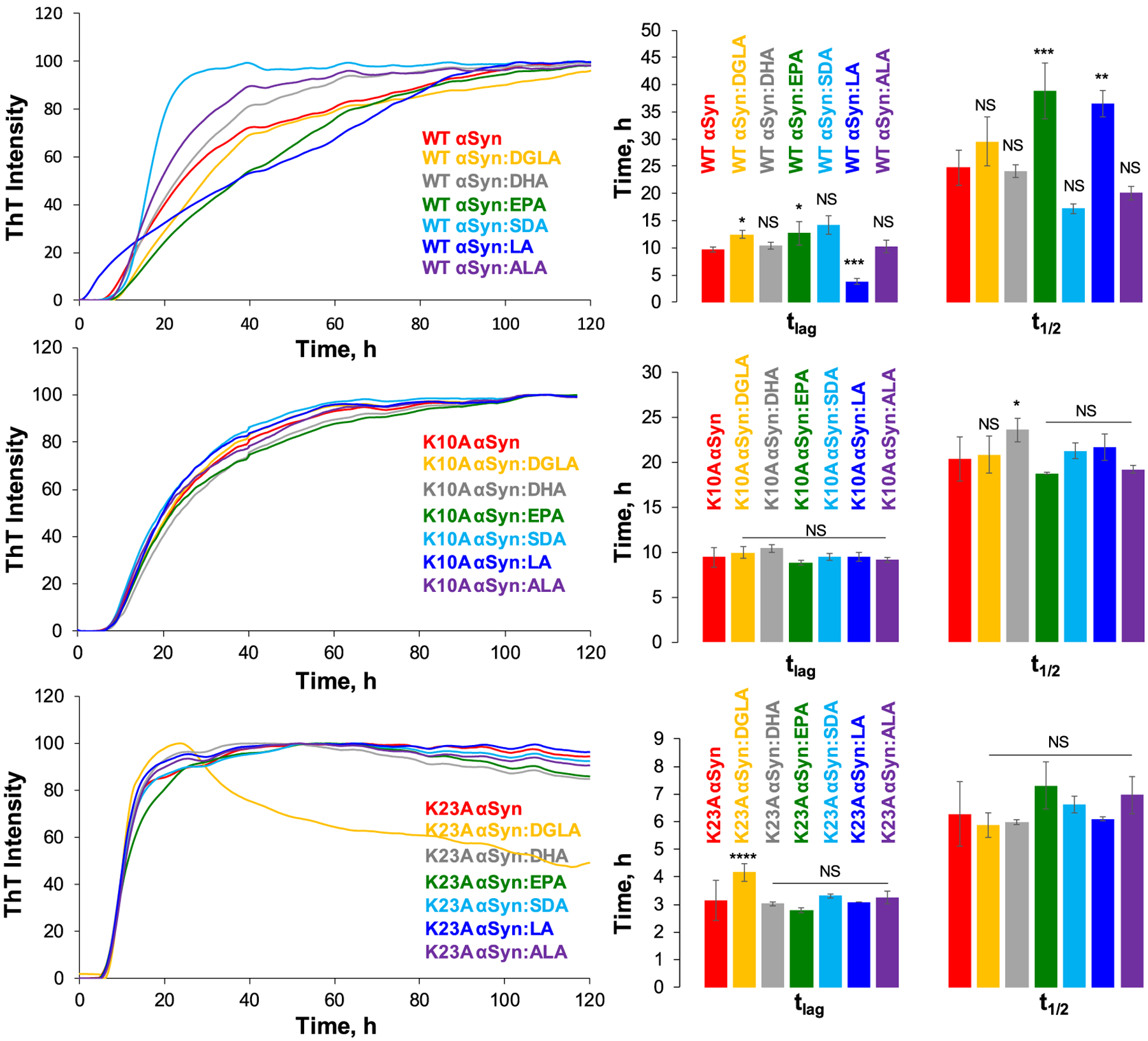
ThT kinetics (left) and corresponding histograms of lag-time (t_lag_) and half-time (t_1/2_) of WT, K10A, and K23A α-syn in the lipid-free environment (red) and in the presence of DGLA (yellow), DHA (grey), EPA (green), SDA (light blue), LA (blue) and ALA (purple); t_lag_ represents 10% increase in the ThT intensity, t_1/2_ represents 50% of maximal ThT intensity. All measurements were made in triplicates. Means of three replicates are shown in the figures. At least two independent experiments were done for each set of samples. One-way ANOVA with Tukey’s honest significant difference post hoc was performed to reveal statistical significance between samples that had no FAs (red) and samples that were incubated in the presence of FAs. NS is a nonsignificant difference; *p ≤ 0.05, **p ≤ 0.01, ***p ≤ 0.001, and ****p ≤ 0.0001.

Our results show that FAs uniquely altered the lag-phase (t_lag_) of WT α-syn aggregation. Specifically, DGLA (t_lag_ = 12.45 ± 0.72 h) and EPA (t_lag_ = 12.67 ± 2.08 h) slightly delayed, whereas LA (t_lag_ = 3.80 ± 0.52 h) drastically shortened t_lag_ of WT α-syn (t_lag_ = 9.72 ± 0.50 h), Fig. 1 and Table S1. No significant changes in t_lag_ of WT α-syn aggregation were observed in the presence of DHA (10.40 ± 0.63), SDA (14.18 ± 1.66) and ALA (10.23 ± 1.08). Based on these results, we can conclude that DGLA, EPA, and LA changed the rate of primary nucleation of WT α-syn, which was reflected in the discussed above differences in t_lags_. We also found that EPA and LA decelerated the elongation of WT α-syn nuclei into fibrils. This conclusion could be made by a significant increase in the half-time (t_1/2_) of WT α-syn aggregation in their presence (Table S1). On the other hand, other PUFAs and SDA did not change the rate of WT α-syn fibril formation.

The ThT assay revealed drastically different behavior of K10A α-syn in the presence of the same FAs. Specifically, none of the analyzed lipids altered t_lag_ of K10A α-syn, Fig. 1 and Table S1. The same conclusion could be made about t_1/2_ except for a small delay in the rate of K10A α-syn aggregation exerted by DHA. Thus, we can conclude that the K10 amino acid residue is critical for α-*syn*-lipid interactions. We observed similar changes in kinetics of K23A and K32A α-syn in the presence of different FAs. Only DGLA (4.16 ± 0.08 and 10.95 ± 0.91) and EPA (2.79 ± 0.08 and 11.30 ± 0.60) delayed t_lag_ of K23A and K32A α-syn, respectively. All other lipids did not cause significant changes in t_lag_ of K23 or K32A α-syn. Furthermore, none of the lipids altered t_1/2_ of K23 and K32A α-syn. Based on these results, we can conclude that, similar to K10, K23 and K32 amino acid residues are critically important for α-*syn*-lipid interactions. As such, their replacements on aliphatic amino acid residues lower FA-induced changes in the aggregation behavior of α-syn mutants.

**Fig. 2.**
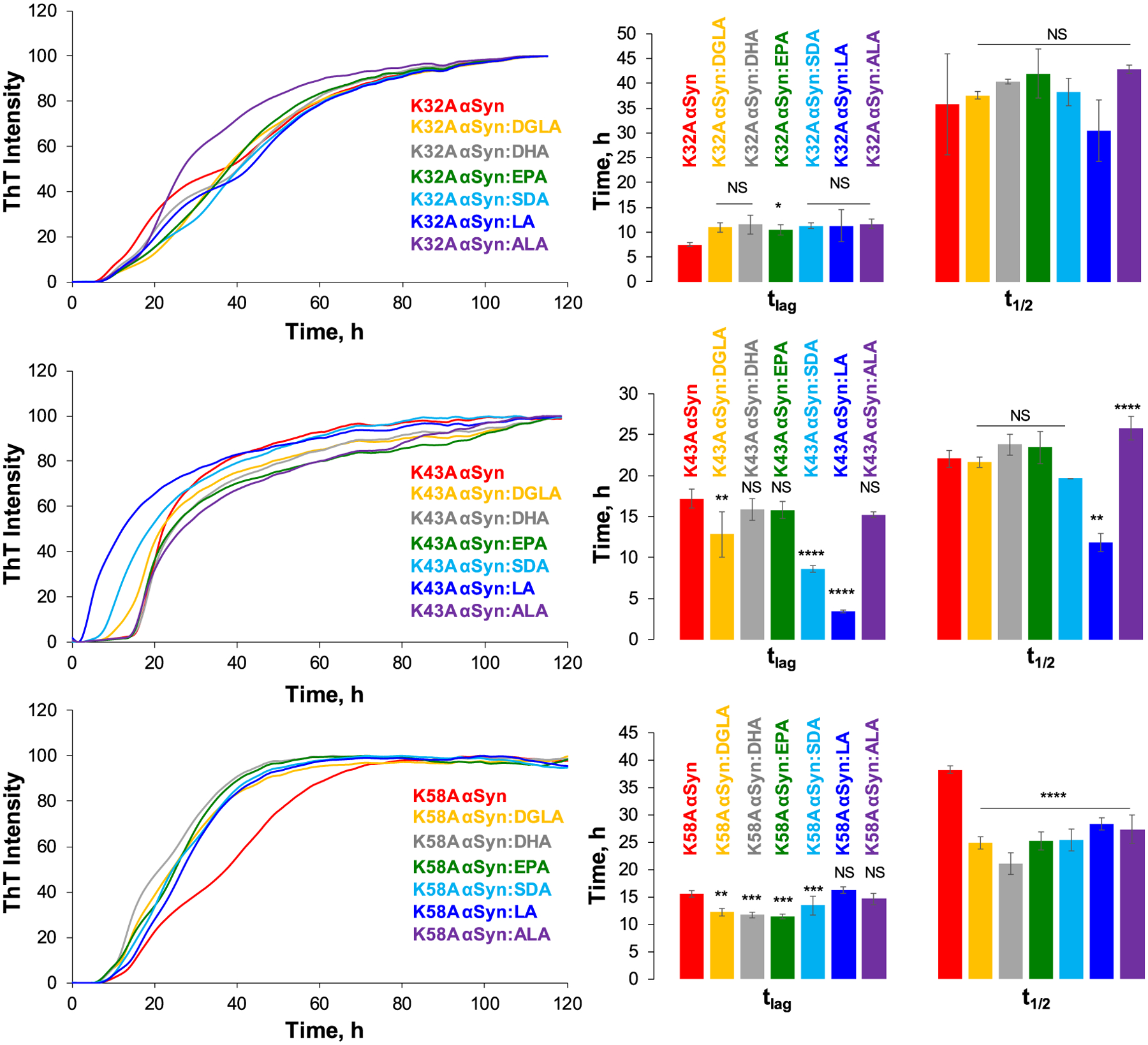
ThT kinetics (left) and corresponding histograms of lag-time (t_lag_) and half-time (t_1/2_) of K32A, K43A, and K58A α-syn in the lipid-free environment (red) and in the presence of DGLA (yellow), DHA (grey), EPA (green), SDA (light blue), LA (blue) and ALA (purple); t_lag_ represents 10% increase in the ThT intensity, t_1/2_ represents 50% of maximal ThT intensity. All measurements were made in triplicates. Means of three replicates are shown in the figures. At least two independent experiments were done for each set of samples. One-way ANOVA with Tukey’s honest significant difference post hoc was performed to reveal statistical significance between samples that had no FAs (red) and samples that were incubated in the presence of FAs. NS is a nonsignificant difference; *p ≤ 0.05, **p ≤ 0.01, ***p ≤ 0.001, and ****p ≤ 0.0001.

The opposite conclusion could be made about K43A and K58A α-syn. We found that DGLA (12.83 ± 2.80), SDA (8.61 ± 0.42), and LA (3.42 ± 0.13) shortened t_lag_ of K43A α-syn aggregation. ALA (11.86 ± 1.12) also increased, whereas LA (25.78 ± 1.46) decreased t_1/2_ of K43A α-syn, Fig. 2. A small decrease in t_lag_ was observed for K58A α-syn in the presence of DGLA (12.20 ± 0.70), DHA (11.69 ± 52), EPA (11.46 ± 0.41), and SDA (13.40 ± 1.70), whereas no changes were found in the presence of LA (16.20 ± 0.63) and ALA (14.66 ± 1.04), Fig. 2. We also found that all analyzed FAs caused a significant increase in t_1/2_ of K58A α-syn aggregation. Thus, we can conclude that FAs alter the aggregation of K43A and K58A α-syn. Based on these results, we can conclude that K43 and K58 amino acids residues are less critical for protein-lipid interactions. Nevertheless, their replacements on aliphatic amino acids possibly alter the secondary structure of α-syn and, consequently, the thermodynamics of α-*syn*-FA interactions, which were reflected by how dissimilar to WT α-syn t_lags_ and t_1/2s_ observed in the presence of different FAs. These findings indicate that protein-lipid interactions are influenced by more than one amino acid and have a more complex nature.

### 2.2. Morphological characterization of K10A, K23A, K32A, K43A, K58A, and WT α-syn formed in the presence of different FAs

**Fig. 3.**
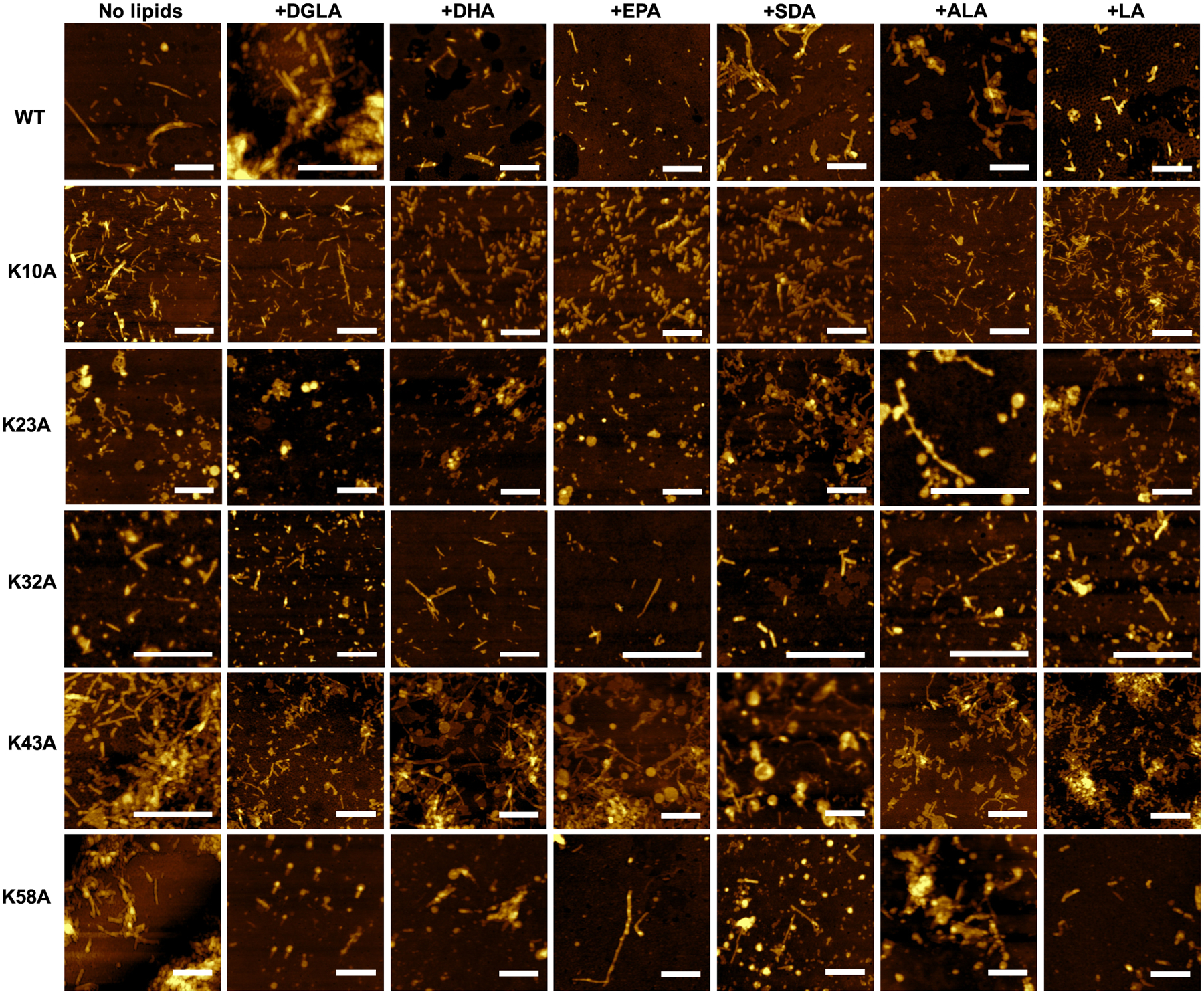
AFM images of WT α-syn, K10A, K23A, K32A, K43A, and K58A α-syn in the lipid-free environment (no lipid) and in the presence of DGLA, DHA, EPA, SDA, LA and ALA. Scale bars are 500 nm. For each AFM image, 3–4 areas of the sample were scanned. At least 20 independent aggregates were measured to obtain height profiles. The images represent the 2 experiments with the 3 technical replicates within each experiment.

**Fig. 4.**
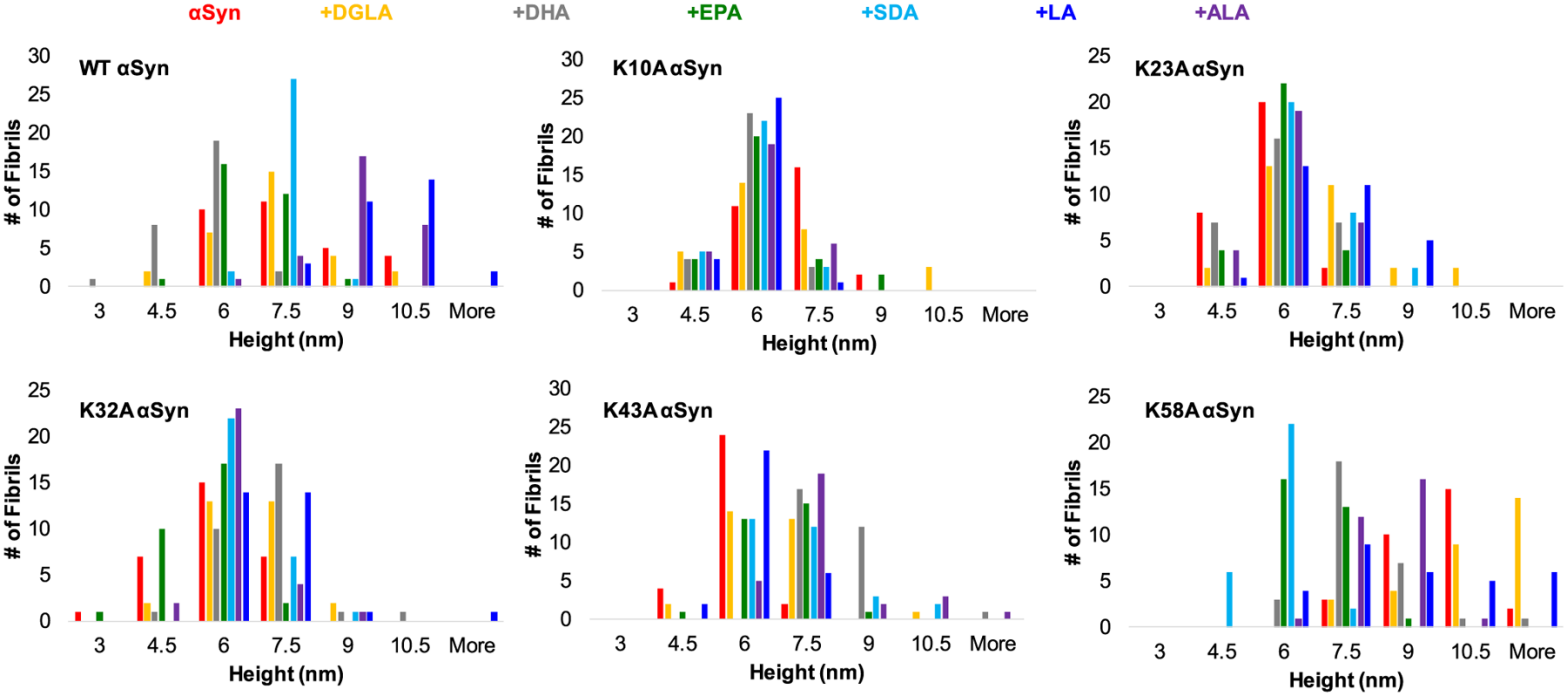
Histograms of heights of WT α-syn, K10A, K23A, K32A, K43A, and K58A α-syn fibrils formed in the lipid-free environment (red) and in the presence of DGLA (yellow), DHA (grey), EPA (green), SDA (light blue), LA (blue) and ALA (purple). For each AFM image, 3–4 areas of the sample were scanned. At least 20 independent aggregates were measured to obtain height profiles. The images represent the 2 experiments with the 3 technical replicates within each experiment.

In the lipid-free environment, WT α-syn formed fibrils with heights ranging from 6 to 10 nm, Figs. 3 and 4. Morphologically similar fibrils were observed in WT α-syn:DGLA, WT α-syn:DHA, and WT α-syn:EPA. However, their height was substantially smaller (4–9 nm) than the height of WT α-syn fibrils formed in the absence of FAs. In the presence of SDA, WT α-syn formed highly uniform fibrils that had ~7.5 nm in height. Finally, the presence of LA and ALA resulted in the formation of thicker fibrils that were 7–10 nm in height. Based on these results, we can conclude that FAs uniquely alter the morphology of WT α-syn fibrils.

We found that K10A, K23A, and K32A α-syn formed morphologically similar if not identical fibrils in the presence of different FAs. Additionally, morphologically, such fibrils were undistinguishable from the aggregates formed by these mutants in the lipid-free environment. All fibril species were, on average, 6 nm in height. In some of the analyzed samples, we also observed lipid droplets co-present with amyloid fibrils. At the same time, the aggregation of K43A α-syn in the presence of different FAs resulted in the formation of fibrils with an average height of 6–7.5 nm. Similar to other samples, some of the observed aggregates were thinner (4.5 nm) or thicker (9–12 nm) than others, Figs. 3 and 4. Finally, K58A α-syn aggregates exhibited similar to WT α-syn variability in heights if aggregated in the presence of different FAs. Specifically, K58A α-syn:SDA fibrils were the thinnest with heights of 4.5–7.5 nm, whereas K58A α-syn:DGLA, K58A α-syn:LA and K58A α-syn fibrils were the thickest with heights ranging from 7.5 to 12 nm. K58A α-syn:DHA and K58A α-syn:EPA fibrils had on average 7.5 nm in height. Based on these results, we can conclude that K10A, K23A, and K32A mutations in α-syn substantially altered protein interactions with FAs which resulted in the formation of morphologically similar, if not identical, fibrils in the presence of different lipids. This effect was less prominent in K43A and K58A α-syn, which indicates that K43 and K58 amino acid residues were less critical for α-*syn*-lipid interactions.

### 2.3. Structural characterization of K10A, K23A, K32A, K43A, K58A, and WT α-syn formed in the presence of different FAs

**Fig. 5.**
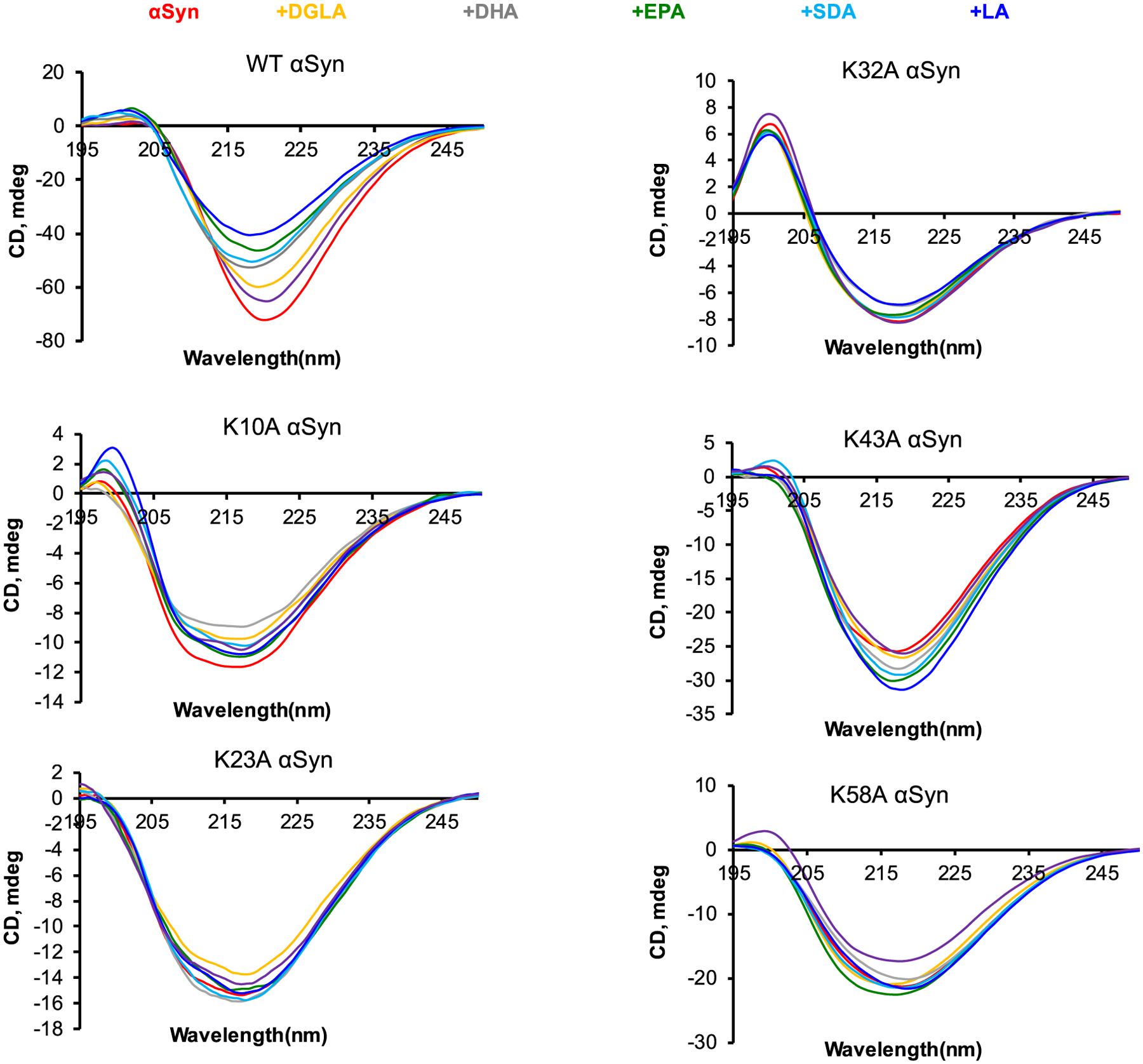
CD spectra acquired from WT α-syn, K10A, K23A, K32A, K43A, and K58A α-syn fibrils formed in the lipid-free environment (red) and in the presence of DGLA (yellow), DHA (grey), EPA (green), SDA (light blue), LA (blue) and ALA (purple). All measurements were made in triplicates. Means of three replicates are shown in the figures. At least two independent experiments were made for each set of samples.

We utilized circular dichroism (CD) and infrared (IR) spectroscopy to determine the secondary structures of K10A, K23A, K32A, K43A, K58A, and WT α-syn fibrils formed in the presence of different FAs. CD spectra acquired from all samples exhibited minima around 250–220 nm, which indicates the predominance of β-sheet in the structure of amyloid aggregates, Fig. 5. Furthermore, CD spectra acquired from different samples of any mutant were highly similar, which indicates that FAs induced very little if any changes in the secondary structure of amyloid fibrils that were formed in their presence. We observed some changes between the CD profiles of different mutants. For example, CD spectra of K32A, K43A, K58A, as well as WT α-syn have narrower minima compared to the spectra acquired from K10A and K23A α-syn that have much broader profiles. One may expect that these spectral differences were caused by the presence of unaggregated protein. The same explanation can be attributed to small variability of CD minima for WT α-syn compared to WT α-syn:DGLA, WT α-syn:DHA, WT α-syn:EPA, WT α-syn:SDA, WT α-syn:LA, and WT α-syn:ALA. To confirm this hypothesis, we utilized atomic force microscopy infrared (AFM-IR) spectroscopy. (Dazzi, 2009; Dazzi et al., 2010; Dazzi et al., 2012)

In AFM-IR, a metallized scanning probe can be positioned at the individual protein aggregate. (Centrone, 2015; Katzenmeyer et al., 2013; Kurouski et al., 2020; Ramer et al., 2017) After that, pulsed tunable IR light is used to induce thermal expansions in the sample. These thermal expansions are passed to the scanning probe and then converted into IR spectra. (Deniset-Besseau et al., 2014; Mathurin et al., 2018; Rebois et al., 2017) In the acquired IR spectra, amide I band can be used to determine the secondary structure of protein aggregates. (Ramer et al., 2018; Ruggeri et al., 2020a; Ruggeri et al., 2021; Ruggeri et al., 2015; Ruggeri et al., 2020b; Ruggeri et al., 2016) Amide *I* centered at ~1630 indicates the presence of parallel β-sheet, whereas the shift of amide I to ~1695 cm^−**1**^ indicates the presence of and anti-parallel β-sheet in the secondary structure of protein aggregates. The presence of amide I at 1660 cm^−**1**^ is evidence for unordered proteins.

**Fig. 6.**
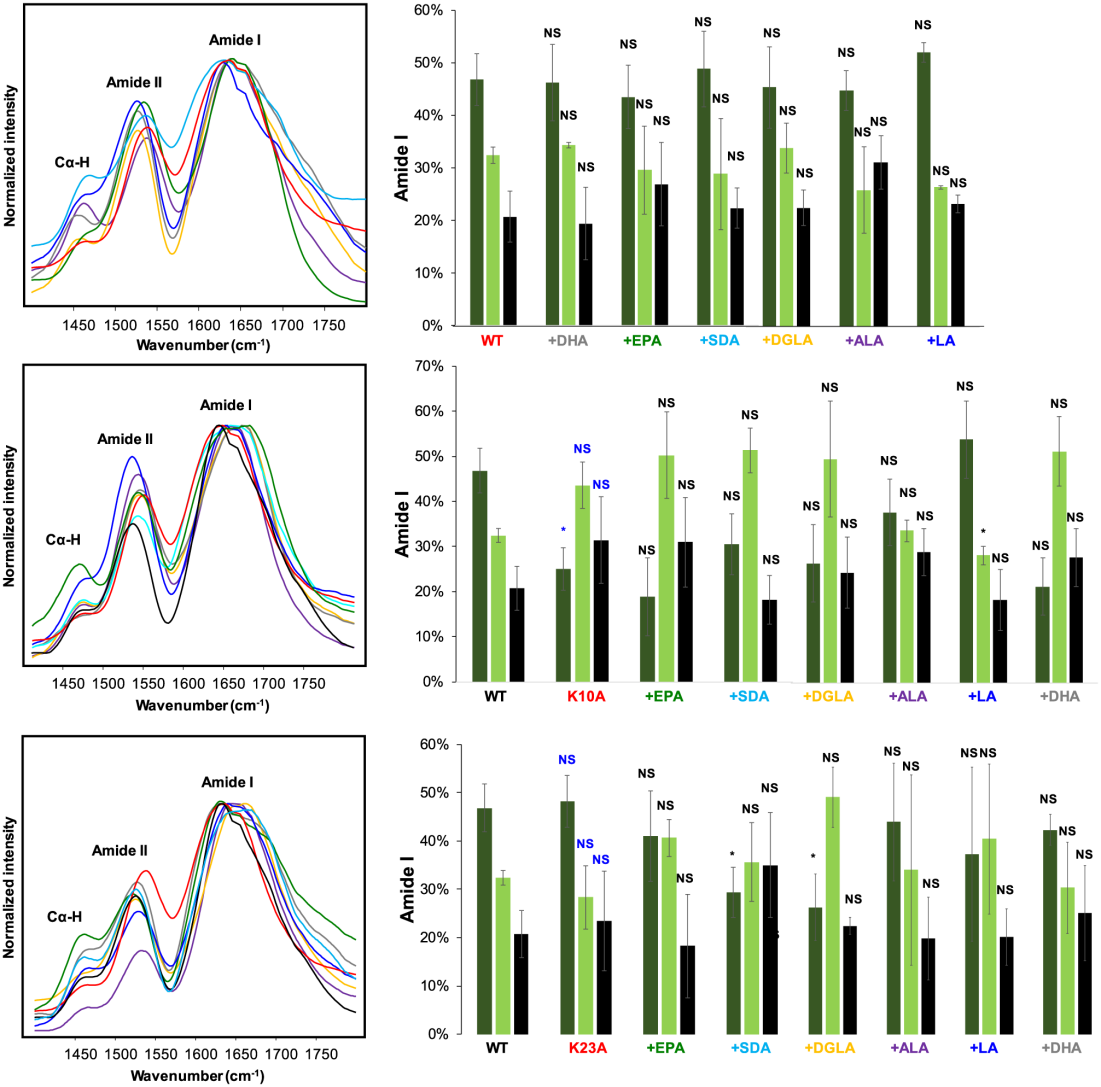
AFM-IR spectra acquired from WT α-syn, K10A, and K23A α-syn fibrils formed in the lipid-free environment (red) and in the presence of EPA (green), SDA (light blue), DGLA (yellow), ALA (purple), LA (blue) and DHA. All measurements were made in triplicates. Means of three replicates are shown in the figures. At least two independent experiments were made for each set of samples. At least 20 different aggregates were analyzed in each sample. One-way ANOVA with Tukey’s honestly significant difference post hoc was performed to reveal statistical significance between samples. Blue “NS” indicates the absence of statistical significance between WT and α-syn mutants grown in the lipid-free environment. Black “NS” is a nonsignificant difference between α-syn mutants grown in the absence and presence of FAs; *p ≤ 0.05, **p ≤ 0.01.

**Fig. 7.**
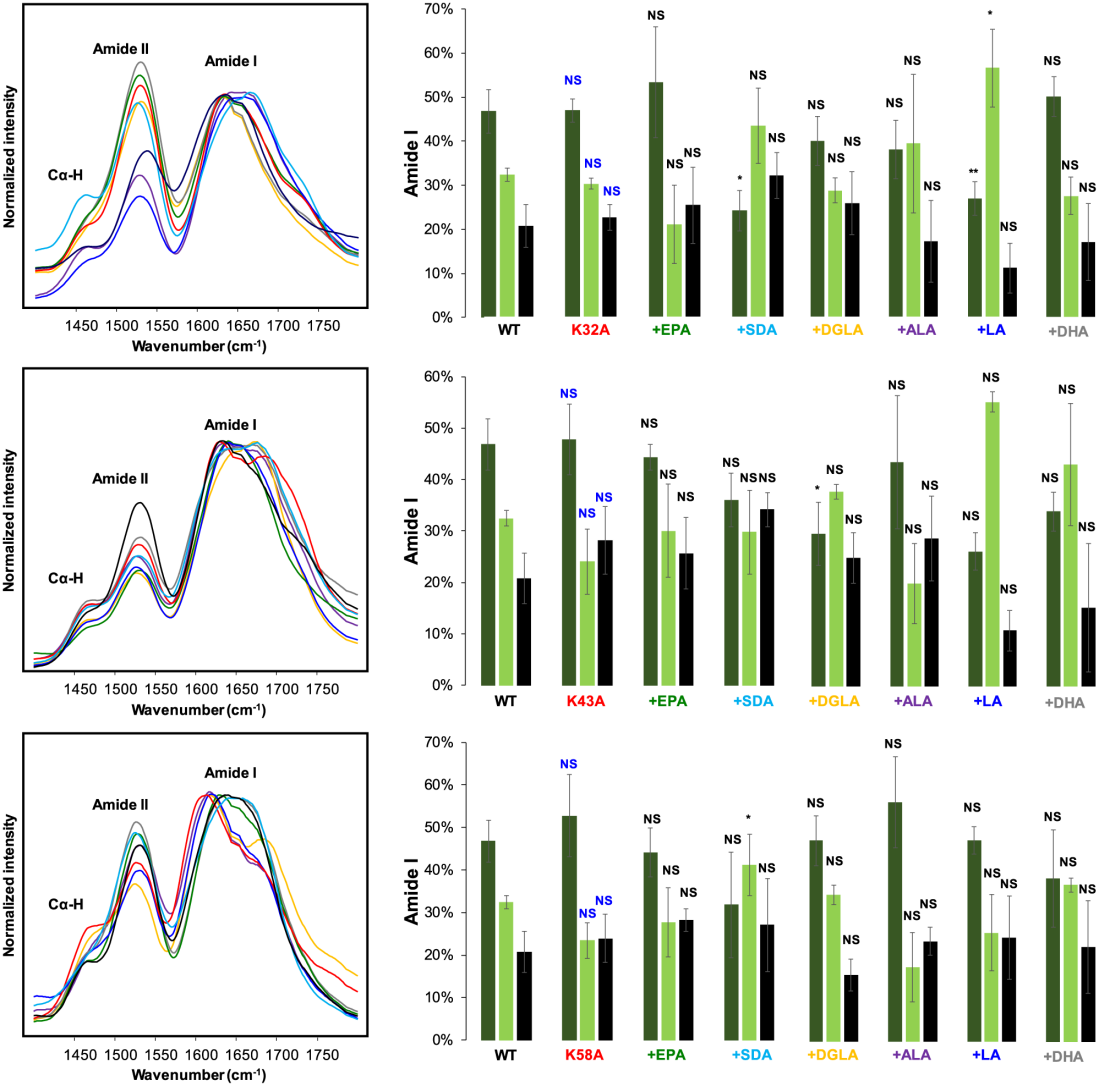
AFM-IR spectra acquired from WT α-syn, K32A, K43A and K58A α-syn fibrils formed in the lipid-free environment (red) and in the presence of EPA (green), SDA (light blue), DGLA (yellow), ALA (purple), LA (blue) and DHA. All measurements were made in triplicates. Means of three replicates are shown in the figures. At least two independent experiments were made for each set of samples. At least 20 different ag-gregates were analyzed in each sample. One-way ANOVA with Tukey’s honestly significant difference post hoc was performed to reveal statistical significance between samples. Blue “NS” indicates the absence of statistical significance between WT and α-syn mutants grown in the lipid-free environment. Black “NS” is a nonsignificant difference between α-syn mutants grown in the absence and presence of FAs; *p ≤ 0.05, **p ≤ 0.01.

AFM-IR revealed no significant differences between the secondary structure of WT α-syn formed in the lipid-free environment and the secondary structure of fibrils formed in the presence of different FAs. All fibrils were dominated by parallel β-sheet (~48%) with a smaller amount of unordered protein (~30%) and antiparallel β-sheet (~22%), Fig. 6. Based on these results, we can conclude that FAs do not alter the secondary structure of WT α-syn fibrils. We also found that K10A α-syn fibrils grown in the lipid-free environment had significantly lower amounts of parallel β-sheet (24%) compared to WT α-syn (~48%). These fibrils had a much higher amount of unordered protein (~45%) and antiparallel β-sheet (~31%), Fig. 6. The secondary structure of K23A α-syn was very similar to the secondary structure of WT α-syn formed in the lipid-free environment. The same conclusion could be made about K23A, K43A, and K58A α-syn grown in the absence of FAs. We also found that only K23A:SDA and K23A:DGLA α-syn had significantly lower amounts of parallel β-sheet in their secondary structure compared to K23A α-syn formed in the lipid-free environment. Similar structural fluctuations were observed for other samples. For instance, K32A:SDA and K32A:LA α-syn fibrils exhibited significantly lower amounts of parallel β-sheet than K32A α-syn fibrils, whereas only K43A:DGLA α-syn had lower amounts of parallel β-sheet than K43A α-syn fibrils, Fig. 7. We found that similar to WT α-syn, FAs did not alter the secondary structure of K58A α-syn fibrils. Based on these findings, we can conclude that changes in the secondary structure of α-syn mutants are unlikely to be linked to K-to-A mutations themselves and rather originate from mutant-specific protein:FA interactions.

### 2.4. Toxicities of K10A, K23A, K32A, K43A, K58A, and WT α-syn formed in the presence of different FAs

**Fig. 8.**
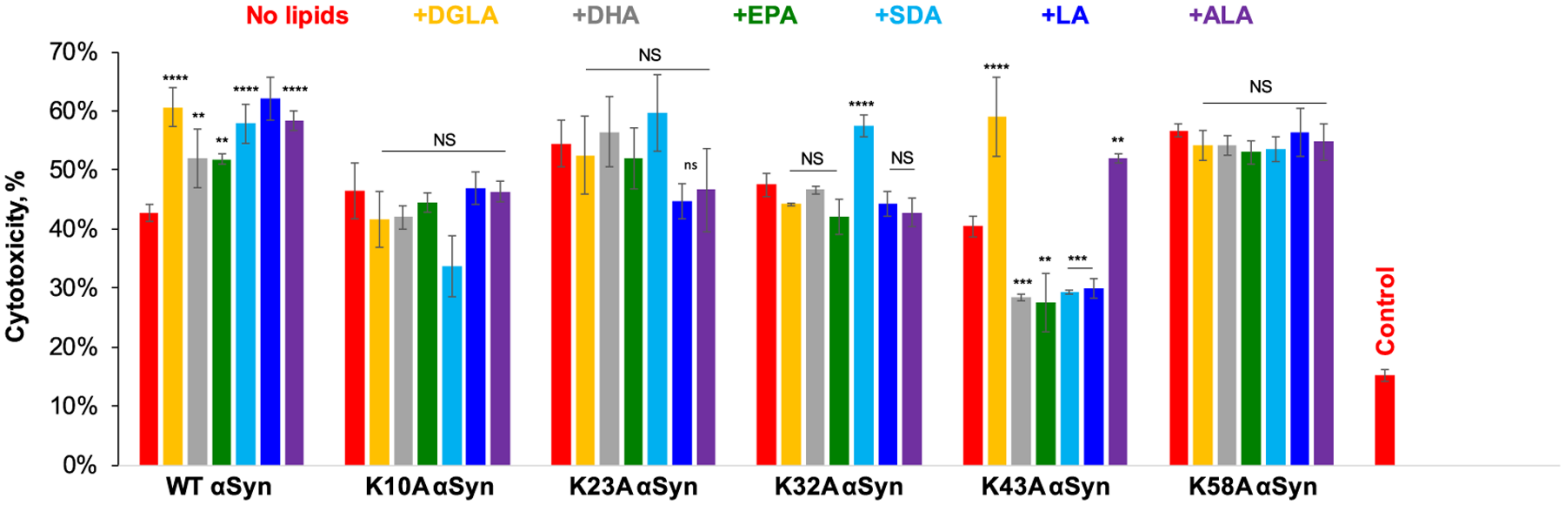
Histograms of LDH assay of WT α-syn, K10A, K23A, K32A, K43A, and K58A α-syn fibrils formed in the lipid-free environment (red) and in the presence of DGLA (yellow), DHA (grey), EPA (green), SDA (light blue), LA (blue) and ALA (purple). All measurements were made in triplicates. Means of three replicates are shown in the figures. At least two independent experiments were made for each set of samples. One-way ANOVA with Tukey’s honestly significant difference post hoc was performed to reveal statistical significance between samples that had no FAs (red) and samples that were incubated in the presence of FAs. NS is a nonsignificant difference; *p ≤ 0.05, **p ≤ 0.01, ***p ≤ 0.001, and ****p ≤ 0.0001.

We used N27 rat dopaminergic neurons to investigate the extent to which different FAs altered the toxicity of K10A, K23A, K32A, K43A, K58A, and WT α-syn fibrils. We found that all analyzed FAs drastically increased the toxicity of WT α-syn fibrils from ~40% (no lipids) to ~50% in the case of DHA and EPA, Fig. 8. Much higher cytotoxicity (~60%) was observed for WT α-syn:DGLA, WT α-syn:SDA, WT α-syn:LA, and WT α-syn:ALA. Based on these results, we can conclude that FAs drastically change the toxicity of WT α-syn fibrils.

The LDH assay showed that K10A and K23A α-syn fibrils formed in the lipid-free environment exerted the same levels of cytotoxicity as their aggregates formed in the presence of different FAs. These findings indicate that FAs do not alter the toxicity of K10A and K23A α-syn fibrils. The same conclusion could be extended to K32A and K58A fibrils. We found that only K32A α-syn:SDA fibrils were found to be more toxic than K32A α-syn fibrils formed in the lipid-free environment.

In the presence of DGLA and ALA, K43A α-syn formed fibrils that exerted higher toxicity than K43A α-syn fibrils formed in the lipid-free environment. Whereas the presence of DHA, EPA, SDA, and LA resulted in the formation of much less toxic than K43A α-syn fibrils. Based on these results, we can conclude that, similar to WT α-syn, FAs uniquely altered the toxicity of K43A α-syn fibrils. Surprisingly, we found that FAs did not alter the toxicity of K58A α-syn fibrils. It should be noted that K58A α-syn fibrils formed in the lipid-free environment exerted much higher toxicity (58%) than WT α-syn (43%) fibrils. Based on these findings, one can expect that high toxicity of K58A α-syn fibrils formed in the lipid-free environment did not allow for the observation of FA-altered toxicity of this mutant. Alternatively, one can expect that the K58 amino acid residue plays an important role in protein-lipid interactions. Although the K58A mutation does not significantly alter kinetics of protein aggregation in the presence of different FAs, as well as morphology of K58A α-syn fibrils, it becomes critically important for lipid-induced changes in the toxicity of α-syn fibrils.

## 3. Discussion

Our study revealed that the WT α-syn stability could be altered by FAs. Furthermore, we found that changes in t_lag_ and t_1/2_ were directly linked to the structure of FAs themselves. These results are in a good agreement with the previously reported results by Ali and co-workers and Holman and co-workers for transthyretin and insulin, respectively. (Holman et al., 2023; Ali et al., 2023b) Remarkably, WT α-syn formed much more toxic fibrils in the presence of FAs, although no significant changes in their secondary structure were observed. Upon analysis of the aggregation properties of the K-to-A mutants, we showed that the effect of FA-induced changes in t_lag_ and t_1/2_ was primarily determined by K10, K23, and K32 amino acid residues. Replacements of these charged amino acid residues with non-polar alanines limited the response of corresponding mutants to FAs that were present at the stage of protein aggregation. These conclusions are further supported by the analysis of morphologies of amyloid fibrils formed by K10A, K23A, and K32A α-syn. AFM imaging showed that all mutants formed the same fibrils in the lipid-free environment and in the presence of different FAs. We also observed no changes in the toxicity of K10A, K23A, and K32A α-syn fibrils grown in the lipid-free environment and in the presence of different FAs. Thus, we can conclude that K10, K23, and K32 play an important role in protein-lipid interactions.

Furthermore, our results show that K43 and K58 amino acid residues of α-syn play a far less important role in protein-lipid interactions. This conclusion could be made based on significant changes in t_lag_ and t_1/2_ of K43A and K58A α-syn observed in the presence of different FAs. As was discussed above, such changes were observed for WT α-syn. AFM imaging also revealed much greater diversity of fibril heights in K43A and K58A α-syn compared to K10A, K23A, and K32A α-syn. Finally, we observed FA-modulated toxicity of K43A α-syn fibrils that were evident for WT α-syn and not detected for K10A, K23A, K32A, and K58A α-syn fibrils. Furthermore, we found that K43A α-syn formed much less toxic fibrils in the presence of DHA, EPA, SDA, and LA compared to the toxicity of both WT and K43A α-syn formed in the lipid-free environment. These results might suggest that these FAs could be used as co-supplements together with possible drugs that will bind to K43 of α-syn inhibiting protein-lipid interactions. Be aware that DGLA and ALA increased the toxicity of K43A α-syn fibrils. These results indicated that protein-FA interactions are fairly complex and require additional studies to fully reveal mechanisms of their interactions.

Holman and co-workers used docking simulations to investigate binding energies of different FAs to insulin. (Holman et al., 2023) The researchers found that FAs with different lengths and saturations could have different energies of interactions with the same protein. These findings help to explain the observation of several outliers in our ThT kinetics. For instance, we found that DGLA and EPA delayed t_lag_ of K23A and K32A α-syn, whereas this effect was not observed for all other FAs. Finally, we want to point out that none of the K-to-A mutations inhibited protein aggregation. These findings indicate that the N-terminus of α-syn does not directly participate in the formation of the fibril core, as was expected based on the previously reported crystal structures of α-syn fibrils, but rather plays an important role in protein-lipid interactions. (Guerrero-Ferreira et al., 2018; Li et al., 2018)

## 4. Conclusions

Site-directed mutagenesis was used to express α-syn mutants that had 5 different K-to-A substitutions in the N terminus of α-syn. Our results showed that K10, K23, and K32 K-to-A replacements minimized or fully disabled interactions of the corresponding mutants with charged FAs. As a result, FAs that were present at the stage of protein aggregation had very little, if any, effect on the rate of primary nucleation and fibril elongation. We also found that the morphology and toxicity of K10A, K23A, and K32A α-syn fibrils formed in the presence of FAs were very similar, if not identical, to fibrils formed by these mutants in the lipid-free environment. However, a completely differentiated behavior of WT α-syn, K43A and K58A α-syn was observed in the presence of the same FAs. We found that FAs with different lengths and saturation uniquely altered the rate of primary nucleation and fibril elongation of these proteins. As a result, WT α-syn, K43A and K58A α-syn fibrils formed in the presence of different FAs had different morphologies. Based on these results, we can conclude that K10, K23, and K32 amino acid residues play an important role in protein-lipid interactions. Whereas K43 and K58 amino acid residues were far less significant for such interactions. Considering a previously reported increase in the toxicity of α-syn fibrils formed in the presence of different lipids, (Dou et al., 2023) one may expect that these findings could be used to develop new molecular drug candidates that will be able to block α-syn interactions with lipids.

## 5. Experimental procedures

### 5.1. Materials

Saturated stearic acid (C18:0, STA) and polyunsaturated linoleic acid (C18:2, LA), α-linoleic acid (C18:3, ALA), dihomo-γ-linolenic acid (C20:3, DGLA), eicosapentaenoic acid (C20:5, EPA), and docosahexaenoic acid (C22:6, DHA) were obtained from Avanti (Alabaster, AL, USA), IPTG was purchased from Sigma-Aldrich (St. Louis, MO, USA).

### 5.2. Cloning and site direct mutagenesis of WT α-syn, and K10A, K23A, K32A, K43A and K58A α-syn

pET-21a αSYN gene fragments served as mutagenesis templates. Site-directed mutations were introduced at specific positions (K10A, K23A, K32A, K43A, and K58A) using designed primers **α-*syn*-K10A-F**-AGCGCAGCGAAAGAAGGCGTG and **α-syn-K10A-R**-GCTG CGCTC AGAC CTTTCATAA. **α-syn-K23A-F**-ACGGCACAGGGCGTGG, **α-syn-K23A-R**-TGTGCCGTTTTT TCCGCC, **α-syn-K32A-F**-GGCGCAACGAAAGAAGGTGT, **α-syn-K32A-R**-GTTGCGCCGG CCGCTTC, **α-syn-K43A-F**-AGCGCAACCAAAGAAGGCG, **α-syn-K43A-R** GTTGCGCTGC CGACATACA, **α-syn-K58A-F-**GAAGCAACGAAAGAACAGGTCAC, **α-syn-K58A-R**- GTT GCTTCTGCAACGGTGGCC. In total, 50 ul PCR reaction was carried out with 50 ng templates, 2 mM primer pair, 200 mM dNTPs, and 2 U of DNA fusion polymerase. The PCR amplification products were evaluated by 1% agarose gel electrophoresis. The PCRs were purified by Pure Link^™^ PCR Purification Kit (Thermo Fisher Scientific Inc) and further treated with restriction enzyme *Dpn*I (NEB). An aliquot of 5 ul above PCR product was transformed into DH5α competent *E. coli* cells and inoculated on Luria–Bertani (LB) plate containing 100 mg/mL ampicillin. A total of 10 colonies were selected and their plasmids were isolated by mini prep (Thermo Fisher Scientific Inc). The positive mutants were selected by respective restriction enzyme (*Nde*I and *Xho*I) digestion. Mutants’ plasmid was sequenced by Eurofin to final confirmation on the mutations.

### 5.3. Protein expression and purification of WT α-syn, and K10A, K23A, K32A, K43A and K58A α-syn

pET21a-alpha-synuclein, as well as K10A, K23A, K32A, K43A, and K58A, was overexpressed in *Escherichia coli BL21 (DE3)* Rosetta strain using LB broth media according to the protocol described by Ali et al. (2023). Two liters of the bacterial culture (1 mM IPTG-induced) were piped down at 8000 RPM for 10 min. The pellet was re-suspended in lysis-tris buffer (50 mM Tris, 10 mM EDTA, 150 mM NaCl, pH 7.5) that contained the protease inhibitor cocktail (Roche); 2 cycle freeze and thaw followed by the sonication. The sonicated sample was boiled in the water bath for 30 min. Next, samples were centrifuged at 16,000 *g* for 30 min and the supernatants were collected. 10% streptomycin sulfate (136 μL/mL) and glacial acetic acid (228 μL/mL) were added to the supernatant followed by centrifugation at 16,000 *g*, 10 min at 4 °C. The resulting supernatant was precipitated by an equal volume of saturated ammonium sulfate at 4 °C. Precipitated samples were washed with (NH_4_)_2_SO_4_ solution at 4 °C (saturated ammonium sulfate and water, 1:1 *v*/v). The washed pellet was then re-suspended using 100 mM NH_4_(CH_3_COO) under constant stirring for 10 min. The protein was precipitated by the addition of an equal volume of absolute ethanol. Ethanol precipitation was repeated twice at room temperature. The collected protein was re-suspended in 100 mM NH_4_(CH_3_COO), lyophilized, and stored at −20 °C for further chromatographic purification.

### 5.4. Size exclusion chromatography (SEC)

WT α-syn, as well as K10A, K23A, K32A, K43A, and K58A α-syn mutants, were dissolved in PBS buffer, pH 7.4. Centrifuged for 30 min at 14,000 g using a benchtop microcentrifuge (Eppendorf centrifuge 5424 USA). Next, 500 μL of WT α-syn, K10A, K23A, K32A, K43A, and K58A α-syn concentrated protein was loaded on a Superdex 200 10/300 gel filtration column in AKTA Pure (GE Healthcare) FPLC. Proteins were eluted isocratically with a flow rate of 0.5 mL/min at 4 °C using the same buffer 1.5 mL fractions were collected according to the UV-VIS detection at 280 nm.

### 5.5. FAs

Stearic acid (C18:0, STA), linoleic acid (C18:2, LA), α-linoleic acid (C18:3, ALA), dihomo-γ-linolenic acid (C20:3, DGLA), eicosapentaenoic acid (C20:5, EPA), and docosahexaenoic acid (C22:6, DHA) were dissolved in phosphate-buffered saline (PBS), pH 7.4. Next, samples were heated in a water bath to ~65 °C for 30 min. After that, samples were immersed in liquid nitrogen for 1 min. The procedure was repeated 8–10 times.

### 5.6. WT α-syn, K10A, K23A, K32A, K43A and K58A α-syn aggregation

In the lipid-free environment, 100 μM of α-syn, K10A, K23A, K32A, K43A and K58A mutants were dissolved in PBS; the solution pH was adjusted to pH 7.4. For DGLA, DHA, SDA, EPA, ALA and LA, 100 μM of α-syn, K10A, K23A, K32A, K43A and K58A mutants were first mixed with an equivalent concentration of the corresponding FAs. Next, the pH of the final solution was adjusted to pH 7.4 using concentrated HCl. After that, samples were placed into 96 well-plate that was kept in the plate reader (Tecan, Männedorf, Switzerland) at 37 °C for 160 h under 510 rpm agitation.

### 5.7. Kinetic measurements

Protein aggregation rates were assessed using a thioflavin T (ThT) fluorescence assay. In this procedure, samples were combined with a 2 mM ThT solution and dispensed into a 96-well plate. The plate was then positioned in a Tecan plate reader (Männedorf, Switzerland) and maintained at 37 °C for 160 h with continuous agitation at 510 rpm. Fluorescence measurements, with excitation at 450 nm and emission at 488 nm, were recorded at 10-min intervals in a Tecan plate reader (Männedorf, Switzerland). Each kinetic curve presented is an average derived from three independent measurements.

### 5.8. AFM imaging

Microscopic analysis of protein aggregates was conducted using an AIST-NT-HORIBA system in Edison, NJ. Silicon AFM probes with a force constant of 2.7 N/m and resonance frequency between 50 and 80 kHz, acquired from Appnano (Mountain View, CA, USA), were employed. The collected AFM images underwent pre-processing using AIST-NT software in Edison, NJ. Solutions of protein and protein-lipid, suspended in a PBS buffer, were diluted in a 1:15 ratio with DI water. For each sample, three 10 × 10 μm areas were analyzed, with 6–7 heights recorded from each before generating the final image.

### 5.9. Circular dichroism (CD)

Following a 160-h incubation of proteins at 37 °C, samples were diluted with PBS and transferred into a quartz cuvette. CD spectra were promptly measured using a Jasco J1000 CD spectrometer from Jasco in Easton, MD, USA. A total of three spectra were collected for each sample across the wavelength range of 190 to 240 nm and subsequently averaged.

### 5.10. Attenuated total reflectance fourier-transform infrared (ATR-FTIR) spectroscopy

Following 160 h of protein aggregation at 37 °C, protein samples were applied to the crystal of a Perkin-Elmer FTIR spectrometer (Waltham, MA, USA) equipped with an ATR module. The deposited samples were allowed to dry at room temperature, and three spectra were acquired from each sample. These spectra were then averaged for further analysis.

### 5.11. AFM-IR

AFM-IR spectra were gathered using a Nano-IR3 system (Bruker, Santa Barbara, CA, USA) equipped with a QCL laser and gold-coated contact-mode AFM scanning probes (ContGB-G AFM probe, Nano-AndMore, Watsonville, CA, USA). The collected spectra underwent averaging three times and were smoothed using a Savitzky-Golay filter (second order) in MATLAB. Subsequently, spectral deconvolution of the averaged spectra was performed in GRAMS/AI, identifying features such as parallel β-sheet at 1624 cm^−1^, α-helix, and random coil at 1655 cm^−1^, and anti-parallel β-sheet at 1695 cm^−1^.

### 5.12. Cell toxicity assays

Cell toxicity assays were performed utilizing the N27 rat dopaminergic neuron cell line. The cells were cultured in 96-well plates with RPMI 1640 Medium supplemented with 10% fetal bovine serum (FBS) at 37 °C and 5% CO_2_. Upon reaching approximately 70% confluency after 24 h of incubation, the cells were prepared for further experimentation.

For the LDH assay, 100 μL of the medium was substituted with RPMI 1640 Medium contain ing 5% FBS and 10 μL of protein samples. The FBS concentration was decreased to reduce the baseline absorbance level of the analyzed samples. Following an additional 24 h of incubation, the CytoTox 96 cytotoxicity assay kit (G1781, Promega, Madison, WI, USA) was employed to quantify the amount of lactate dehydrogenase (LDH) released into the cell culture medium. LDH, an enzyme present in the cytosol, is released upon damage to the plasma membrane.

LDH concentration was determined by measuring the conversion of lactate to pyruvate through NAD+ reduction to NADH. This reduction facilitated the conversion of a tetrazolium salt to a red formazan product with an absorption maximum at 490 nm. The level of formazan produced directly correlated with the amount of LDH released, offering a measure of the toxicity of the protein aggregates to the N27 cells.

## Supplementary Material

SI

Appendix A. Supplementary data

Supplementary data to this article can be found online at https://doi.org/10.1016/j.nbd.2024.106553.

## Data Availability

Data will be made available on request.
