# Supplementary material for: Elucidating the mechanisms of α-Synuclein-lipid interactions using site-directed mutagenesis
Source: Neurobiol Dis. Author manuscript; Available in PMC 2025 Jun 11. (PMC12152419; doi:10.1016/j.nbd.2024.106553)
Supplement: SI [file NIHMS2082084-supplement-SI.docx]

**Elucidating the Mechanisms of α-Synuclein-Lipid Interactions Using Site-Directed Mutagenesis.**

Abid Ali^1^, Aidan P. Holman^1,2^, Axell Rodriguez^1^, Luke Osborne^1^, and Dmitry Kurouski^1,3*^

1. Department of Biochemistry and Biophysics, Texas A&M University, College Station, Texas 77843, United States

2. Department of Entomology, Texas A&M University, College Station, Texas 77843, United States

3. Department of Biomedical Engineering, Texas A&M University, College Station, Texas, 77843, United States

Email: [dkurouski@tamu.edu](mailto:dkurouski@tamu.edu)

Supporting Information:

Figure S1. Chemical structures of FAs used in the study.

Table S1. ThT kinetics corresponding lag-time (t_lag_) and half-time (t_1/2_) of WT, K10A, K23A, K32A, K43A, and K58A α-syn in the lipid-free environment and in the presence of different FAs.

|  | t_lag_ | t_1/2_ |
| --- | --- | --- |
| aSYN | 9.72±0.50 | 24.73±3.24 |
| aSYN:DGLA | 12.45±0.72 | 29.56±4.47 |
| aSYN:DHA | 10.40±0.63 | 24.05±1.21 |
| aSYN:SDA | 14.18±1.66 | 17.17±0.83 |
| aSYN:EPA | 12.67±2.08 | 38.89±5.11 |
| aSYN:ALA | 10.23±1.08 | 20.07±1.32 |
| aSYN:LA | 3.80±0.52 | 36.55±2.45 |
| K10A | 9.44±1.08 | 20.41±2.43 |
| K10A:DGLA | 9.97±0.65 | 20.82±2.43 |
| K10A:DHA | 10.43±0.43 | 23.60±1.33 |
| K10A:SDA | 8.83±0.24 | 18.79±0.13 |
| K10A:EPA | 9.48±0.33 | 21.29±0.91 |
| K10A:ALA | 9.51±0.48 | 21.68±1.48 |
| K10A:LA | 9.17±0.23 | 19.219±0.41 |
| K23A | 3.13±0.32 | 6.27±0.42 |
| K23A:DGLA | 4.16±0.08 | 5.87±0.08 |
| K23A:DHA | 3.02±0.08 | 5.99±0.36 |
| K23A:SDA | 3.30±0.08 | 6.61±0.29 |
| K23A:EPA | 2.79±0.08 | 7.30±0.84 |
| K23A:ALA | 3.25±0.24 | 6.96±0.66 |
| K23A:LA | 3.08±0.00 | 6.10±0.08 |
| K32A | 7.47±0.35 | 35.78±10.15 |
| K32A:DGLA | 10.95±0.91 | 37.49±0.77 |
| K32A:DHA | 11.52±1.89 | 40.29±0.44 |
| K32A:SDA | 10.44±1.09 | 41.94±4.90 |
| K32A:EPA | 11.30±0.60 | 38.23±2.80 |
| K32A:ALA | 11.30±3.28 | 30.47±6.17 |
| K32A:LA | 11.64±0.91 | 42.85±0.85 |
| K43A | 17.17±1.17 | 22.07±1.00 |
| K43A:DGLA | 12.83±2.80 | 21.62±0.63 |
| K43A:DHA | 15.85±1.32 | 23.78±1.21 |
| K43A:SDA | 8.61±0.42 | 19.68±0.00 |
| K43A:EPA | 15.80±1.05 | 23.44±1.94 |
| K43A:ALA | 15.23±0.36 | 25.78±1.46 |
| K43A:LA | 3.42±0.13 | 11.86±1.12 |
| K58A | 15.57±0.60 | 38.22±0.70 |
| K58A:DGLA | 12.20±0.70 | 24.87±1.05 |
| K58A:DHA | 11.69±52 | 21.11±2.04 |
| K58A:SDA | 13.40±1.70 | 25.44±1.96 |
| K58A:EPA | 11.46±0.41 | 25.21±1.69 |
| K58A:ALA | 14.66±1.04 | 27.38±2.61 |
| K43A:LA | 16.20±0.63 | 28.29±1.12 |
